# Deciphering the potential role of PGRN in regulating CD8^+^ T cell antitumor immunity

**DOI:** 10.1038/s41420-024-02001-7

**Published:** 2024-05-14

**Authors:** Wenyu Zhang, Huan Qin, Guosheng Wang, Jing Zhang, Wenjuan He, Chunmei Feng, Huimin Wan, Feilong Wang, Zhongliang Guo

**Affiliations:** 1 Department of Pulmonary and Critical Care Medicine, Shanghai East Hospital, School of Medicine, Tongji University, Shanghai, 200092 China; 2 Medical College, Tongji University, Shanghai, 200092 China

**Keywords:** Non-small-cell lung cancer, Cancer microenvironment, Immunosuppression, Cancer therapeutic resistance, Cancer immunotherapy

## Abstract

A key factor contributing to resistance in immune checkpoint blockade (ICB) therapies is CD8^+^ T-cell tolerance in the tumor microenvironment (TME), partly resulting from upregulating coinhibitory receptors. Here, we describe the role of PGRN as a coinhibitory molecule that modulates the antitumor response of CD8^+^ T cells, thus presenting a novel immunosuppressive target for lung cancer. The in vivo subcutaneous transplanted lung cancer model showed that PGRN expression was elevated on CD8^+^ T cells that infiltrated transplanted lung cancers. Furthermore, PGRN deficiency was found to specifically encourage the infiltration of CD8^+^ T cells, enhance their proliferation, migration, and activation, and resist apoptosis, ultimately inhibiting tumor growth. This was achieved by PGRN knockout, increasing the production of T cell chemokine CCL3, which boosts the antitumor immune response induced by CD8^+^ T cells. Critically, the PD-L1 inhibitor exhibited a synergistic effect in enhancing the antitumor response in PGRN^–/–^ mice. In summary, our findings highlight the significance of PGRN as a novel target for boosting CD8^+^ T cells antitumor immunity and its potential to overcome the resistance in ICB therapy.

## Introduction

Immune checkpoint blockade (ICB)-based immunotherapy, which includes treatments of anti-programmed death 1 (PD-1) as well as anti-programmed death-ligand 1 (PD-L1), has significantly transformed the treatment landscape for lung cancer by reactivating dormant antitumor T-cell effector responses [1, 2]. However, most patients do not respond to single-agent α-PD-1/PD-L1 treatments due to primary or acquired resistance to the therapy [3]. In these cases, PD-1 signaling does not act as the limiting factor in the cancer immunity cycle, and blocking PD-1 or PD-L1 alone is insufficient to restore antitumor immunity [4]. Other immune checkpoints, immunosuppressive immune cells, abnormal angiogenesis, and cytokines also play a role in setting the cancer-immune balance and promoting immune tolerance [5–7]. Therefore, addressing these negative factors could improve the effectiveness of α-PD-1/PD-L1 therapy and overcome drug resistance.

Evidences emphasize that the cross-talk between CD8^+^ T cells as well as tumor cells defines the tumor’s immune status, promoting or impeding ICB effectiveness. Generally, a higher number and enhanced function of infiltrating CD8^+^ cytotoxic T cells (CTL) within the tumor correlate with a better response to ICB [8, 9]. However, CD8^+^ CTL cells exert potent antitumor functions while also being induced by TME to gradually develop immune tolerance, leading to the expression of high levels of inhibitory checkpoints (namely CTLA-4, PD-1, LAG-3, and TIM-3) in addition to reduced effector functions. These factors contribute to acquired resistance to ICB [10, 11]. Therefore, overcoming acquired resistance should concentrate on alleviating multiple inhibitory mechanisms and rejuvenating exhausted CD8^+^ T cells, fostering a more robust immune response. The combined application of multiple ICBs has also shown higher clinical efficacy. For instance, clinical trials have demonstrated that combining ipilimumab (anti-CTLA-4) and nivolumab (anti-PD-1) shows superior efficacy compared to standard targeted therapy, chemotherapy, or monotherapies with ipilimumab or nivolumab [12–17]. Presently, this treatment regimen—ipilimumab with nivolumab—has been authorized by the FDA for melanoma, PD-L1-positive NSCLC, in addition to malignant pleural mesothelioma [12, 18]. Various dual immune checkpoint blockade strategies, some of which are FDA- or NMPA- approved, while others are still in clinical trials, include combinations such as α-PD-1/PD-L1 with α-TIM-3, α-Siglec-15, and α-LAG-3. In early-phase trials in the ICB-resistant scenario, inhibiting these targets showed positive safety and activity results, either alone or in combination with PD-1 or PD-L1 inhibitors [19–21]. Therefore, we are dedicated to identifying a novel targeting molecule that can not only restore the active function of CD8^+^ T cells, breaking the T-cell immune tolerance induced by the TME but also develop new combination anti-PD-1/PD-L1 treatment strategies for improved efficacy.

Progranulin (PGRN), a secreted immunomodulatory protein with 593 amino acid residues, is associated with several characteristics in tumor cells, such as malignancy, invasiveness, and susceptibility to radiochemical agents [22]. Recent studies have identified its crucial role in mediating immune evasion in tumors and implicated it as a potential therapeutic target. Fang et al. discovered that PGRN promotes the polarization of M2-type macrophages and upregulates PD-L1 expression to maintain an immunosuppressive state in the TME of breast cancer [23]. Cheung et al. discovered that PGRN promotes immune evasion in pancreatic ductal adenocarcinoma by promoting autophagy-dependent MHCI degradation [24]. Nonetheless, the expression pattern and function of PGRN are cancer-specific [25]. In lung cancer, only Chen et al. reported significantly high expression of PGRN in lung cancer tissues and investigated the relationship between PGRN and the malignancy of the tumor cells [26]. Its impact on tumor immunity during lung cancer development, especially its comprehensive role in T cell-mediated antitumor responses and immunotherapy, remains unexplored.

In this study, we utilized mice lung cancer models to clarify PGRN as an inhibitory molecule that regulates T-cell antitumor responses and its potential as a target in combination immunotherapy strategies for lung cancer. Initially, we observed increased PGRN expression in CD8^+^ tumor-infiltrating lymphocytes (TILs) in a subcutaneous Lewis lung cancer model. Subsequent research revealed that PGRN knockout enhanced the host antitumor immunity, suppressed tumor growth, and upregulated chemokine CCL3 expression in the CD8^+^ T cells. This promotes CD8^+^ T cell recruitment and accumulation within the TME and enhances their proliferation, activation, and cytotoxicity while delaying exhaustion, resulting in improved antitumor immune response. Our findings indicated that PGRN ablation plus PD-L1 blockers have synergistic antitumor effects in mice with lung cancer. This combination therapy exhibited superior antitumor efficacy by augmenting CD8^+^T cell infiltration and functionality in the TME. In summary, this study offers innovative therapeutic strategies and credible preclinical data for translating this novel combination therapy into clinical practice for lung cancer immunotherapy.

## Results

### PGRN deficiency enhances CD8^+^ T cell infiltration and function within the TME in vivo

**Fig. 1:**
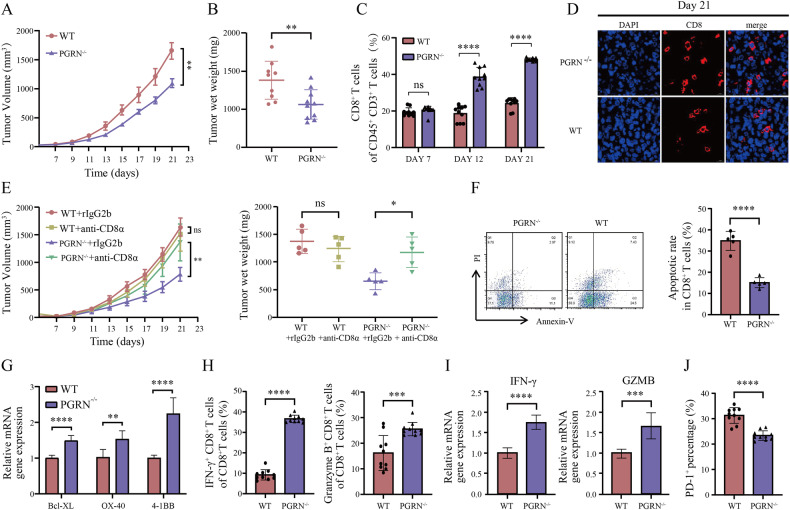
PGRN knockout improves CD8^+^ T-cell infiltration and function in TME. **A** PGRN-KO and WT mice received subcutaneous injections of 1 × 10^6^ Lewis cells. The volume of tumor transplants was monitored by measuring them every two days, leading to the construction of growth curves (mean ± SEM; *n* = 10/group; **p < 0.01). **B** All subcutaneously transplanted tumors were subjected to tumor weight measurements (*n* = 10/group, **p < 0.01). **C** The proportions of CD8^+^ T cells within the CD45^+^CD3^+^ cell population in tumor tissues on days 7, 12, and 21 following the subcutaneous injection of tumor cells into mice (mean ± SD; *n* = 10/group; ****p < 0.0001). **D** Representative immunofluorescence staining with CD8 (red) and DAPI (blue) in tumor tissues on day 21 following the subcutaneous injection of tumor cells into mice; scale bar = 50 μm. **E** CD8^+^ T cell neutralization assays: PGRN-KO and WT mice were subcutaneously injected with 1 × 10^6^ Lewis cells. Upon reaching an average tumor volume of 100 mm^3^, mice received intraperitoneal injections of rat IgG2b isotype or anti-CD8 neutralizing antibody every three days for four injections. The measurement of volumes for subcutaneously transplanted tumors was performed every two days. The resulting growth curves and tumor weights are presented (mean ± SEM; *n* = 8/group; *p < 0.05, **p < 0.01, ns nonsignificant). **F** CD8^+^ TILs were observed within the tumors of both WT and PGRN-KO mice and subsequently subjected to staining for an apoptosis assay. The flow cytometry technique was then utilized to determine the proportion of apoptotic CD8^+^ TILs (mean ± SD; *n* = 5/group; ****p < 0.0001). **G** RT-PCR analysis of mRNA levels of specific genes in CD8^+^ T cells sorted by FACS from tumors on day 21 post-tumor inoculation in WT and PGRN-KO mice. Statistical analyses were conducted through the utilization of a t-test (*n* = 3; **p < 0.01; ****p < 0.0001). **H** CD8^+^ T cells isolated from tumor tissues of WT and PGRN-KO mice were stimulated for 6 h with Leukocyte Activation Cocktail (containing the phorbol ester, PMA, a calcium ionophore, and a protein transport inhibitor), followed by flow cytometry to assess cytotoxicity markers of the cell (namely IFN-γ and GZMB) (*n* = 10/group; ***p < 0.001, ****p < 0.0001). **I** RT-PCR analysis of mRNA levels of IFN-γ and GZMB in CD8^+^ T cells sorted by FACS from tumors on day 21 post-tumor inoculation in WT and PGRN-KO mice. Statistical analyses were conducted through the utilization of a t-test (*n* = 3; ***p < 0.001; ****p < 0.0001). **J** Exhaustion marker levels of PD-1 on CD8^+^ T cells within the tumor tissues of WT and PGRN-KO mice were assessed using flow cytometry (*n* = 10/group; ****p < 0.0001).

For investigating the effect of PGRN on the antitumor immunity in vivo, we constructed PGRN^–/–^ mice on a C57BL/6 genetic background (Supplementary Fig. S1A). These mice demonstrated no defects in growth, development, fertility, body weight, or behavior (data not shown). Subsequently, Lewis cells underwent subcutaneous injection into the right flanks of age- and sex-matched WT and PGRN^–/–^ mice, and tumor progression was monitored. As displayed in Fig. 1A, tumors in PGRN^–/–^ mice exhibited significantly reduced volume and weight in comparison to those in WT mice (Fig. 1A, B and Supplementary Fig. S1B). The tumor’s immune landscape was analyzed using flow cytometry, revealing that PGRN ablation caused a substantial rise in the number of CD8^+^ T cells within TME. However, other immune cell types involving NK cells, CD4^+^ T cells, and dendritic cells, in addition to M1 and M2 macrophages, showed no statistically significant changes (Supplementary Fig. S1C). Furthermore, we observed a progressive increase in tumor tissue infiltration by the CD8^+^ T cells in PGRN^–/–^ mice over time (Fig. 1C), as evidenced by immunofluorescence analysis (Fig. 1D and Supplementary Fig. S1D). Such findings indicate that PGRN knockout results in enhanced CD8^+^ T cell tumor infiltration and reduced lung tumor growth in mice.

To investigate the essential role of CD8^+^ T cells in controlling the growth of Lewis tumors in PGRN^–/–^ mice, we initiated CD8^+^ T cell depletion using a specific antibody during tumor development. In WT mice, no significant difference existed in tumor growth between CD8^+^ T cell depletion and rIgG2b-treated groups. However, in PGRN^–/–^ mice, CD8^+^ T cell depletion significantly blocked the inhibitory impact caused by PGRN knockout on tumor growth (Fig. 1E). These results confirm the critical contribution of CD8^+^ T cells to the controlled tumor growth in PGRN^–/–^ mice. Additionally, we conducted RT-PCR to assess PGRN expression in CD8^+^TILs isolated from the tumors and CD8^+^ T cells residing in the spleen on day 21 post-implantation of Lewis tumors into WT mice. Intriguingly, PGRN messenger RNA (mRNA) levels were significantly upregulated in CD8^+^ T cells from tumors than that from spleen, indicating the impact of PGRN on modulating the contribution of tumor-specific CD8^+^ T cells (Supplementary Fig. S2A). Furthermore, our investigation revealed that PGRN expression on CD8^+^TILs was significantly higher than that on infiltrating CD4^+^TILs and macrophage cells in the TME of WT tumor-loaded mice, further confirming the significant impact of PGRN on CD8^+^ TILs in the TME (Supplementary Fig. S8A).

To exclude the impact of PGRN expression by Lewis tumor cells on the TME in PGRN^–/–^ mice, we first compared the expression of PGRN in Lewis cells with that in infiltrating CD4^+^, CD8^+^ T cells and macrophages in the TME of WT tumor-bearing mice. We found that Lewis cells expressed much less PGRN compared to the infiltrating immune cells (Supplementary Fig. S8A). Additionally, we also examined the expression of PGRN in tumor tissue from WT and PGRN^–/–^ mice. As shown in Supplementary Fig. S8B, the level of PGRN was significantly lower in tumor tissue isolated from PGRN^–/–^ mice than that from control mice, indicating that Lewis cells are not the major contributor of PGRN expression in the TME. This supports the validity of the Lewis tumor model in PGRN^–/–^ mice.

To investigate the mechanism underlying PGRN^–/–^ CD8^+^ T cell accumulation in the Lewis lung cancer model, flow cytometry was employed to evaluate the susceptibility of the CD8^+^ TILs from WT and PGRN^–/–^ mice to apoptosis. Figure 1F displays a reduced CD8^+^ TIL apoptosis ratio in the PGRN^–/–^ group in comparison with that in the WT group on day 21, subsequent to tumor inoculation. Moreover, we analyzed the mRNA expression levels of the pro-survival co-stimulatory receptors OX-40 [27] and 4-1BB [28], along with the anti-apoptotic factor Bcl-XL [29], in CD8^+^ TILs (Fig. 1G), revealing upregulated expression of such genes from PGRN^–/–^ mice. These findings indicate that PGRN negatively affects the survival of CD8^+^ TIL-infiltrating tumors and that PGRN knockout contributes to the build-up of this particular subset within the tumor. CD8^+^ T cells are crucial in antitumor immunity, effectively eliminating tumor cells via releasing cytotoxic molecules, namely IFN-γ and GZMB [30]. Accordingly, an analysis of expression levels of these cytotoxicity markers in CD8^+^ TILs was conducted to assess the impact of PGRN on their functional competence. As Fig. 1H illustrates, our results revealed a substantial increase in IFN-γ and GZMB expressions in the CD8^+^ TILs of the PGRN^–/–^ group in comparison with the WT group, as confirmed by RT-PCR analysis (Fig. 1I). This observation further demonstrates the regulatory role of PGRN in modulating CD8^+^ T cell activation.

Research has shown that CD8^+^ T cell-infiltrating tumors progressively undergo a state of “exhaustion”, characterized by upregulated inhibitory receptors (PD-1) and reduced effector functions (cytokine production and cytotoxic capability) [31, 32]. Accordingly, an evaluation of the exhaustion status of CD8^+^ TILs was performed, revealing a notable decrease in the expression of PD-1 on PGRN^–/–^CD8^+^ TIL cells compared with the WT group (Fig. 1J). These findings indicate that PGRN knockout enhances the antitumor immunity induced by the CD8^+^ T cells by increasing the quantity and functionality of CD8^+^ T cells in the TME while alleviating their exhaustion.

### PGRN-deficient CD8+ T cells exhibit enhanced effector function and decreased exhaustion in vitro

**Fig. 2:**
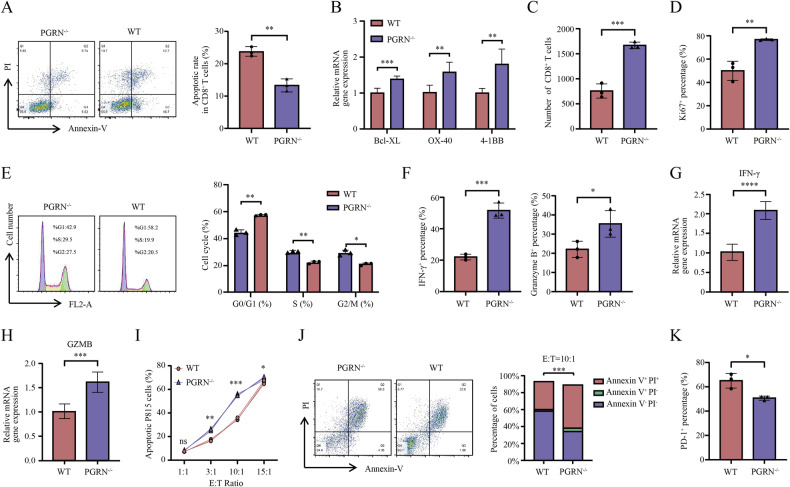
PGRN knockout augments the immune response against tumors and delays the onset of exhaustion in vitro in CD8^+^ T cells. **A** WT as well as PGRN-KO CD8^+^ T cells underwent staining for an apoptosis assay in an in vitro culture model. The flow cytometry technique was deployed to identify the number of apoptotic CD8^+^ T cells (*n* = 3/group; **p < 0.01). **B** The mRNA levels of specific genes underwent analysis using RT-PCR in WT as well as PGRN-KO CD8^+^ T cells. Statistical analyses were conducted through the utilization of a t-test (*n* = 3; **p < 0.01; ***p < 0.001). **C** CD8^+^ T cell count that transversed the transwell system membrane was quantified (*n* = 3/group; mean ± SD; ***p < 0.001). **D** Ki67 staining was used to determine the percentages of proliferating CD8^+^ T cells using flow cytometry (*n* = 3/group; mean ± SD; **p < 0.01). **E** WT and PGRN-KO CD8^+^ T cells were stained for a cell cycle analysis. The flow cytometry technique was utilized to assess the number of CD8^+^ T cells at different stages of the cell cycle (*n* = 3/group; *p < 0.05, **p < 0.01). **F** WT as well as PGRN-KO CD8^+^ T cells underwent stimulation for 6 h by employing Leukocyte Activation Cocktail, and then the flow cytometry analysis was deployed to assess cytotoxicity markers of the cell (namely, IFN-γ and GZMB) (*n* = 3/group; *p < 0.05, ***p < 0.001). **G** IFN-γ mRNA levels in WT and PGRN-KO CD8^+^ T cells were examined using RT-PCR. Statistical analyses were conducted through the utilization of a t-test (*n* = 3/group; ****p < 0.0001). **H** GZMB mRNA levels in WT and PGRN-KO CD8^+^ T cells (*n* = 3/group; ***p < 0.001). **I** P815 cells underwent co-culture with pre-activated CD8^+^ T cells under different ratios. After a 24-h incubation, apoptotic P815 cell percentage was determined using flow cytometry in an apoptosis assay (*n* = 3/group; *p < 0.05, **p < 0.01, ***p < 0.001). **J** P815 cells underwent co-culture with pre-activated CD8^+^ T cells at (1:10 ratio/24 h). The percentage of P815 cells in different apoptotic stages was assessed using flow cytometry (*n* = 3/group; ***p < 0.001). **K** Exhaustion marker levels of PD-1 on WT and PGRN-KO CD8^+^ T cells were assessed using the flow cytometry technique (*n* = 3/group; *p < 0.05).

To comprehensively examine PGRN’s impact on CD8^+^ T-cell activity and function, we established an in vitro CD8^+^ T-cell culture model. Subsequent to their isolation, naive CD8^+^ T cells underwent purification from the spleen, ensuring a purity exceeding 99%, as evidenced by flow cytometry (Supplementary Fig. S2B). Such naive CD8^+^ T cells underwent culture in a 24-well plate pre-coated with CD3 antibody, followed by activation using full media containing CD28 antibody and IL-2. After 72-h stimulation, we conducted an apoptosis flow assay on CD8^+^ T cells, revealing a significant reduction in apoptotic rates for PGRN^–/–^ CD8^+^ T cells compared with the WT group, consistent with our RT-PCR experiments, indicating that PGRN knockout reduces the susceptibility of CD8^+^ T cells to apoptosis (Fig. 2A, B). Moreover, in vitro migration assays demonstrated that PGRN knockout enhanced the migration capabilities of CD8^+^ T cells (Fig. 2C). Remarkably, PGRN^–/–^ CD8^+^ T cells exhibited elevated Ki67 expression and a substantial elevation in cell proportion in G0/G1 phase in comparison with WT CD8^+^ T cells, demonstrating that PGRN knockout promotes CD8^+^ T cell proliferation (Fig. 2D, E).

For investigating the influence of PGRN knockout on the cytotoxicity of CD8^+^ T cells, we assessed the expression of cytotoxic markers after stimulation with CD3-CD28 antibody and IL-2 in vitro. PGRN^–/–^ CD8^+^ T cells presented higher IFN-γ and GZMB expression than WT CD8^+^ T cells, demonstrating that PGRN knockout enhances CD8^+^ T cell activation (Fig. 2F–H). Subsequently, in vitro analysis of CD8^+^ T cell killing activity was deployed by co-culturing the cells with mouse tumor cells. The results revealed that PGRN^–/–^ CD8^+^ T cells displayed stronger killing activity in an E:T ratio-dependent manner than WT CD8^+^ T cells, with a peak difference at an E:T ratio of 10:1 and a tumor cell-killing rate of approximately 1.5 times greater at that time (Fig. 2I, J). Additionally, we observed notably reduced expression of exhaustion marker PD-1 on the surface of PGRN^–/–^ CD8^+^ T cells (Fig. 2K). Collectively, such findings indicate that PGRN knockout increases CD8^+^ T cell-mediated antitumor immunity while delaying their exhaustion.

### PGRN deficiency increases the expression of the T cell chemokine CCL3

To uncover the molecular mechanisms that govern the inhibitory impact of PGRN on CD8^+^ T-cell function and its impact on CD8^+^ T-cell recruitment in the TME, we conducted RNA sequencing analysis. We found that 2569 genes were upregulated, and 2937 were downregulated in PGRN^–/–^ CD8^+^ T cells in comparison with WT CD8^+^ T cells (q < 0.05, log2 ≥ 1.0) (Supplementary Fig. S2E). Consistent with our previous findings, we observed elevated expression levels of IFN-γ, GZMB, and TNF-α in PGRN^–/–^ group (Supplementary Fig. S4A, B). Moreover, genomic enrichment analysis revealed substantial enrichment of cytokine-cytokine receptor interaction pathways in CD8^+^ T cells following PGRN knockout (Supplementary Fig. S4C, D), including eight chemokines with more than two-fold enrichment. Subsequently, qRT-PCR confirmed the upregulation of mRNA levels of CCL3, CCL4, CCL1, CCL9, and CXCL16 in CD8^+^ T cells after PGRN knockout. Notably, CCL3 exhibited the most substantial increase, and this upregulation was verified using western blotting (Supplementary Fig. S4E–G). Prior research has disclosed the pivotal function of CCL3 in mediating migration and recruitment of T and dendritic cells to the TME [33–35] in addition to activation and cytolytic activity of CD8^+^ T cells [36–38]. As an adjuvant therapy, exogenous CCL3 not only augments recruitment and activity of CD8^+^ T cells within the TME but also promotes generation of tumor-specific T cell memory, thereby enhancing antitumor immunity [39]. Consequently, we propose that CCL3 may serve as a key downstream regulatory molecule of PGRN in regulating CD8^+^ T cells.

### PGRN deficiency promotes antitumor immunity in the lung cancer through CCL3

**Fig. 3:**
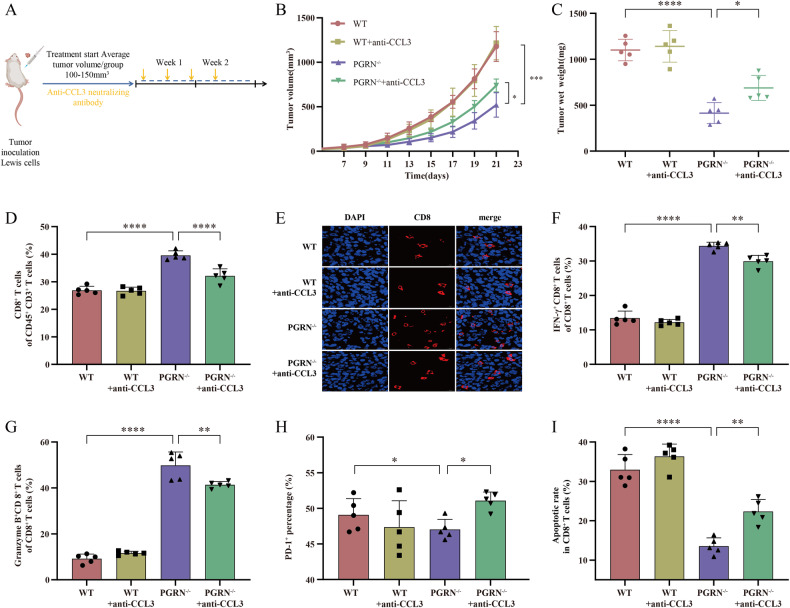
CCL3-mediated enhanced in vivo antitumor immunity caused by PGRN knockout. **A** The treatment schedule for anti-CCL3 neutralizing antibody. **B** The measurements of tumor volume were conducted every two days, leading to the construction of growth curves (mean ± SEM; *n* = 5/group; *p < 0.05, ***p < 0.001). **C** In four different groups of mice, tumor weights were measured for subcutaneously transplanted tumors (*n* = 5/group, *p < 0.05, ****p < 0.0001). **D** The percentages of CD8^+^ T cells within the CD45^+^CD3^+^ cell population in tumor tissues were evaluated using the flow cytometry technique (*n* = 5/group; ****p < 0.0001). **E** Representative immunofluorescence staining with CD8 (red) and DAPI (blue) in tumor tissues from four mouse groups; scale bar = 50 μm. Flow cytometry was utilized to identify (**F**) IFN-γ, (**G**) GZMB, and (**H**) PD-1 expression in CD8^+^ T cells in all groups (*n* = 5/group; **p < 0.01, ****p < 0.0001 for IFN-γ and GZMB; *p < 0.05 for PD-1). **I** Using flow cytometry analysis, the percentage of apoptosis was determined among CD8^+^ T cells infiltrated in tumor tissue (*n* = 5/group; **p < 0.01; ****p < 0.0001).

For investigating the function of CCL3 in the immune response of PGRN-mediated CD8^+^ T cells, Lewis cells underwent subcutaneous injection into WT and PGRN^–/–^ mice. When the tumor volumes reached 100 mm^3^, we divided these mice into subgroups and administered the treatment plan outlined in Fig. 3A. The anti-CCL3 neutralizing antibody did not affect the tumor growth of the WT group but partially eliminated the inhibitory effect on tumor growth induced by PGRN knockout (Fig. 3B, C). We then performed flow cytometry and immunofluorescence analyses to explore the immune landscape within the tumors of each group. The results revealed that the anti-CCL3 neutralizing antibody reduced the infiltration and functional activity of CD8^+^ T cells while accelerating exhaustion (Fig. 3D–H) and increased the apoptotic ratio in PGRN^–/–^ group in comparison with WT group (Fig. 3I).

To validate the findings, we sorted CD8^+^ T cells from PGRN^–/–^ and WT mice and cultured them in vitro to assess the impact of the anti-CCL3 neutralizing antibody. Supplementary Fig. S5A–C display that the antibody significantly raised the apoptosis rate and reduced the expression levels of the cytotoxicity markers (namely IFN-γ and GZMB) in PGRN^–/–^ CD8^+^ T cells without impacting WT CD8^+^ T cells. Moreover, the percentage of PD-1^+^ CD8^+^ T cells in the PGRN^–/–^ group increased (Supplementary Fig. S5D). Besides, the antibody decreased the PGRN^–/–^ CD8^+^ T cell proportion in the proliferative phase, reduced Ki67 expression (Supplementary Fig. S5E, F), and partially blocked the enhanced migration of the CD8^+^ T cells, which is induced by PGRN knockout (Supplementary Fig. S5G). Finally, using the T-cell killing assay, we discovered that the potent cytotoxicity of PGRN^–/–^ CD8^+^ T cells was partially reduced when treated with the anti-CCL3 neutralizing antibody (Supplementary Fig. S5H). Such results disclose that PGRN knockout boosts the antitumor immunity of CD8^+^ T cells in a CCL3-dependent manner.

### PD-L1 blockers induce a synergistic antitumor response in PGRN^–/–^ mice

**Fig. 4:**
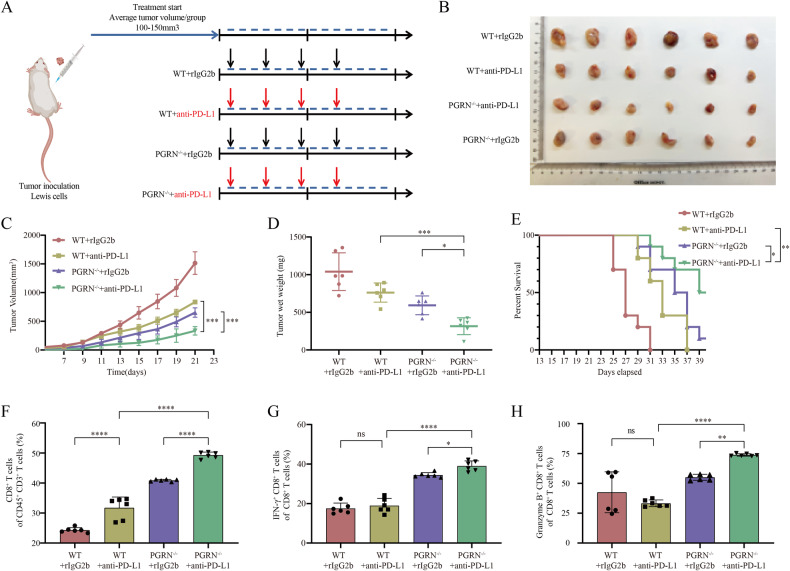
PD-L1 blockade synergistically inhibits tumor growth in PGRN^–/–^ mice. **A** The treatment strategy. **B** The images of tumors from the four treatment groups. **C** Tumor volumes of all tumors transplanted subcutaneously were recorded every two days, resulting in the construction of growth curves (mean ± SEM; *n* = 6/group; ***p < 0.001). **D** The tumor weights were measured for all subcutaneously transplanted tumors (*n* = 6/group, *p < 0.05, ***p < 0.001). **E** Kaplan–Meier analysis of the treatment groups. Flow cytometry analyses in subcutaneously transplanted tumors of treatment for (**F**) CD8^+^ T cell percentages within the CD45^+^CD3^+^ cell population (*n* = 6/group; ****p < 0.0001), **G** IFN-γ^+^ CD8^+^ T cells (*n* = 6/group; *p < 0.05, ****p < 0^.^0001)^,^ in addition to (**H**) GZMB^+^ CD8^+^ T cells (*n* = 6/group; **p < 0.01, ****p < 0.0001; ns nonsignificant).

Our previous findings suggested that PGRN knockout enhanced expression levels of the chemokine CCL3 in CD8^+^ T cells, leading to greater CD8^+^ T-cell infiltration into the TME and improved functional activity. This is noteworthy because the extent of CD8^+^ T-cell infiltration and functionality in TME is well-documented to be positively correlated with the efficacy of ICB therapy [8, 40]. Consequently, we investigated whether tumors in PGRN^–/–^ mice exhibited improved antitumor response to PD-L1 pathway blockade. To evaluate this, we established subcutaneous Lewis lung cancer models in WT and PGRN^–/–^ mice and administered ICB or rIgG2b to distinct groups (Fig. 4A). The results demonstrated that the anti-PD-L1 antibody significantly decreased tumor growth while improving overall survival rates in PGRN^–/–^ mice compared with WT mice (Fig. 4B–E). Regarding the tumor immune landscape on day 21 post antibody treatment, flow cytometry revealed that the anti-PD-L1 antibody had a synergistic effect with PGRN ablation, resulting in more intratumoral CD8^+^ T cells as well as CD8^+^ CTLs (IFN-γ^+^/GZMB^+^ CD8^+^ T cells). Conversely, in WT mice, the anti-PD-L1 displayed a stronger antitumor effect than rIgG2b and improved the extent of CD8^+^ T cell infiltration within the TME without a significant difference in the proportion of cytotoxic CD8^+^ T cells between the two groups (Fig. 4F–H). These results illustrate that PD-L1 inhibitors can synergize with PGRN ablation to improve CD8^+^ T cell-induced antitumor responses and effectively restrain lung tumor growth in mice.

## Discussion

Much research demonstrates the presence of multiple checkpoint axes in the exhausted TILs, referred to as “compensatory upregulation of immune checkpoint inhibitors”. This dictates that blocking one immune checkpoint molecule upregulates other exhaustion markers [41]. Such a rationale may account for the limited efficacy exhibited by single-agent anti-PD-1/PD-L1 therapeutics. Overcoming this resistance mechanism includes combining current ICBs with novel checkpoint inhibitors or other therapeutic approaches to accomplish a synergistic impact that surpasses the sum of individual components [42]. Currently, several combination therapies are being developed with promising results [12–21].

**Fig. 5:**
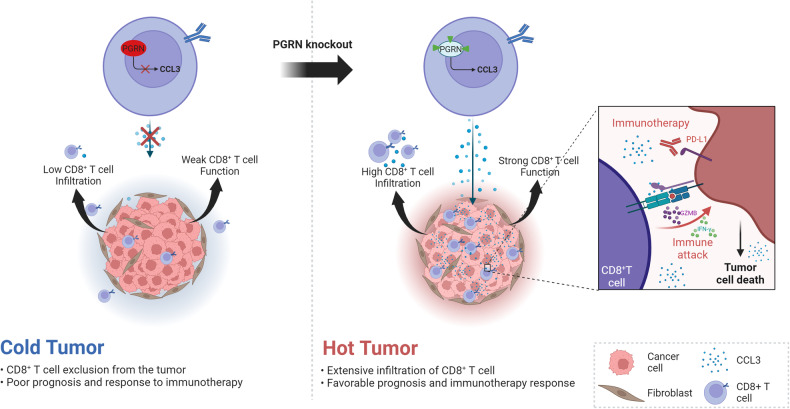
Schematic illustration of the function of PGRN in lung cancer antitumor immunity. Left: abnormally elevated PGRN levels in CD8^+^ T cells contribute to reduced CD8^+^ T-cell infiltration and function in TME due to suppressed CCL3 expression, leading to a “cold” tumor state resistant to immunotherapy. Right: PGRN knockout in CD8^+^ T cells promotes CCL3 production and improves CD8^+^ T-cell infiltration and function in TME, transforming the tumor into a “hot” state, enhancing sensitivity to immunotherapy, and restraining tumor growth.

In this study, we utilized PGRN-deficient mice to elucidate PGRN’s role in regulating antitumor T cell responses. We found that PGRN expression was induced upon activation in CD8^+^ TILs. Subsequently, enhanced effector function in PGRN^–/–^ CD8^+^ T cells, in comparison to WT CD8^+^ T cells, revealed the immune T-cell checkpoint activity of PGRN. Moreover, PGRN^–/–^ mice significantly inhibited the growth of transplanted lung cancers, primarily due to boosted effector function of tumor-infiltrating CD8^+^ T cells. Additionally, we observed that PGRN^–/–^ mice treated with anti-PD-L1 antibodies displayed the most effective tumor control in addition to the longest survival periods. Immune monitoring analysis of the TME revealed that using anti-PD-L1 antibody further increased both the quantity and functionality of CD8^+^ T cells within the TME of PGRN^–/–^ mice. This augmentation enhances the PD-L1-mediated antitumor response of CD8^+^ T cells, significantly reducing tumor growth. These findings suggest that targeting PGRN in combination with other checkpoints, such as PD-L1, may offer a novel therapeutic approach to disrupt immune tolerance of the CD8^+^ T cells in the TME, thereby enhancing the effectiveness of anti-PD-L1 immunotherapy (Fig. 5). Collectively, our study employed a preclinical mouse model of lung cancer to introduce a novel therapeutic approach aimed at improving sensitivity and surpassing resistance to anti-PD-L1 immunotherapy.

Additionally, RNA-seq was used to explore the mechanisms by which PGRN regulates CD8^+^ T cell activation and function. The results revealed a substantial enrichment of cytokine-cytokine receptor and chemokine signaling pathways following PGRN knockout. Chemokines and their receptors are critical in regulating immune cell trafficking and shaping the immune landscape within tumors [43]. We found that PGRN knockout induced the most significant upregulation in chemokine CCL3 expression, primarily produced by immune cells [44]. CCL3 is widely recognized for its close association with CD8^+^ TILs in terms of their infiltration and function within tumors [33–35]. Moreover, activated CD8^+^ TILs release increased amounts of CCL3, further attracting leukocytes and amplifying immune responses [45, 46]. Notably, applying CCL3 neutralizing antibodies partially eliminated the alterations in the antitumor function of CD8^+^ T cells resulting from PGRN knockout. Consequently, we conclude that PGRN knockout upregulates CCL3 expression, leading to enhanced CD8^+^ T-cell infiltration and functional activity within TME, ultimately resulting in the inhibition of tumor growth.

Interestingly, the PGRN confirmed in this article may regulate CD8^+^ T cells by mediating the expression of chemokine CCL3, which is similar to another classic immune checkpoint, SIGLEC15 [20]. Similar expression patterns of PGRN and CCL3 were observed in CD8^+^ T cells isolated from the peripheral blood of patients with lung cancer. Specifically, PGRN expression by CD8^+^ T cells from peripheral blood mononuclear cells (PBMCs) was significantly higher in lung cancer patients compared to healthy individuals, whereas CCL3 levels were significantly reduced (Supplementary Fig. S6). These data suggest that the model described in the mouse system where PGRN regulates CD8^+^ T cells by modulating CCL3 expression may also occur in human lung cancer patients. Based on analysis across tumor types, PGRN exhibits a synchronous expression trend (positive correlation) with other conventional immune checkpoints. In most cancer types, the infiltration of PGRN and CD8^+^ T cells exhibits a predominantly negative correlation, while it demonstrates a positive correlation with certain immunosuppressive immune cells, such as M2 macrophages (Supplementary Fig. S7). The observed synchronous expression pattern between classical immune checkpoints and its influence on immune infiltration across various tumor types indicates that PGRN may serve as a promising novel target for stimulating immune activity.

However, our study had some limitations. First, although our experiments demonstrated the role of PGRN in immune evasion in lung cancer by regulating CCL3 release by CD8^+^ T cells and their cytotoxicity, the complex mechanisms must be further investigated. Second, this investigation overlooked the effect of PGRN on other tumor-infiltrating cell types, sch as macrophages regarding CCL3 secretion, which should be investigated in future studies. Third, to validate the synergistic antitumor effects of in vivo dual therapy targeting PGRN and anti-PD-L1, it is necessary to develop small-molecule inhibitors of PGRN in future research.

## Materials and methods

### Cell lines and cell culture

The Lewis mouse lung cancer cell line, supplied by the American Type Culture Collection, was subjected to testing to guarantee the absence of mycoplasma or any other pathogens. This cell line was cultivated in DMEM, a Gibco product sourced from the USA, which was supplemented with 10% fetal bovine serum in addition to 100 IU/mL of penicillin-streptomycin solution, also from Gibco. The temperature of the cultures was preserved at 37 °C in a humidified incubator that contained 5% CO_2_.

### Mice

Six-to-eight-week-old mice, either PGRN^–/–^ or C57BL/6 (wild-type (WT) controls), supplied by Shanghai Model Organisms Center Ltd., were accommodated in an animal facility under specific pathogen-free (SPF) settings. Typically, PGRN^–/–^ mice were generated at Shanghai Model Organisms Center Ltd. and maintained under SPF conditions. The Institutional Animal Care and Use Committees at Tongji University approved these experiments with approval number TJBB03723105.

### Patient samples

All blood samples were collected following a protocol approved by the Medical Ethics Committee of East Hospital, Tongji University (approval number: 2023-106), after obtaining written informed consent from the patients.

### Antibodies and reagents

Supplementary Table S1 lists the employed reagents and antibodies in this study.

### Tumor model and treatment regimes

WT and PGRN^–/–^ mice—aged 6–8 weeks—received subcutaneous injections of 1 × 10^6^ Lewis cells. The measurements of tumor volume were conducted every two days through the utilization of digital calipers, employing the subsequent equation: volume (*V*)= length (*L*) × width (*W*)^2^/2. Once the tumors in the mice averaged around 50 mm³ in volume, random assignment of the mice into specified study groups was accomplished. Treatment plans were conducted once the average tumor volume reached 100–150 mm^3^ in all groups. In CD8^+^ T cell depletion experiments, each mouse received intraperitoneal injections of 250 μg anti-CD8 neutralizing antibody. For PD-L1 blockade, each mouse was intraperitoneally injected with an anti-PD-L1 monoclonal antibody at a dosage of 10 mg/kg. Anti-CCL3 neutralizing antibody was intraperitoneally administered to mice at a dosage of 250 ng per mouse. All agents were administered once every three days for four times.

### Flow cytometry

To generate single-cell suspensions, diced tumor tissues were processed with a tumor dissociation kit. These suspensions were subjected to staining with various reagents. For identifying dead cells, Fixable Viability Stain 700 was used; T cells were labeled using the following antibodies: anti-CD45, anti-CD3, anti-CD4, in addition to anti-CD8; M2-macrophages were marked by anti-CD11b, anti-F4/80 and anti-CD206 antibodies; M1-macrophages were marked by anti-CD11b, anti-F4/80 and anti-CD86 antibodies; MDSCs were labeled with anti-CD11b, anti-LY6G, and anti-LY6C; and NK cells were tagged with anti-NK1.1. The markers of the CD8^+^ T-cells (namely Ki67, IFN-γ, GZMB, and PD-1) underwent staining as well. The procedure for staining cell surface markers entailed a 30-min incubation in the darkness at 4 °C. Intracytoplasmic markers (IFN-γ and GZMB) and intra-nuclear markers (Ki67) were stained following membrane rupture, with a 40-min fixation period. Apoptosis and cell cycle analyses were performed using the Apoptosis Kit and Cell Cycle Assay Kit, respectively, in a dark environment at 4 °C. Samples for fluid analysis underwent processing through a BD-LSRFortessa flow cytometer. In contrast, those intended for fluid sorting were subject to examination utilizing a BD-FCASAria III flow cytometer. Detailed information regarding the reagents and antibodies is provided in Supplementary Table S1.

### Immunofluorescence (IF)

Resected tumor tissues were washed with PBS, embedded in an optimal cutting temperature compound, and frozen for cryosections. For IF staining, the primary antibody used was anti-CD8 before the use of a secondary antibody, Cy3-conjugated Goat anti-rabbit IgG. For nuclei visualization, DAPI staining was applied. Microscopic analysis of the IF images was performed using an ECLIPSE C1 Ortho-Fluorescent Microscope (Nikon, Japan). Further information regarding the antibodies used is available in Supplementary Table S1.

### Isolation and culturing of CD8^+^ T cells

Following the EasySep Mouse CD8^+^ T cell isolation kit instructions, we isolated naive CD8^+^ T cells from mouse spleens, resuspended them in the specified medium, and placed them in a 24-well plate pre-coated overnight with the CD3 antibody (5 μg/mL). Supplementation with CD28 antibody at 2 μg/mL in addition to IL-2 at 20 ng/mL facilitated activation, leading to the execution of functional assays 72 h later.

### CD8^+^ T cell-mediated cytotoxicity assay

The P815 cell line expresses fragment crystallizing gamma receptor II (FcγRII) on its membrane surface. This receptor enables binding to the Fc region of mouse IgG antibodies, which, via their fragment antigen binding (Fab), can recognize T-cell activation receptors, resulting in cell lysis. P815 cells underwent centrifugation, a washing process, and subsequent resuspension in the culture medium, reaching a concentration of 5 × 10^5^ cells/mL before being seeded in 24-well culture plates (1 mL/well) and incubated overnight. The following day, the activated CD8^+^ T cells were introduced into the upper chamber of the co-culture system at a specified ratio, with a total volume of 300 μL, using the upper layer of the co-culture wells as a template. After 18-h co-incubation, P815 cells from the lower chamber were harvested and labeled with apoptotic markers, with the apoptotic cell ratio being quantified via flow cytometry (Supplementary Fig. S2D).

### In vitro CD8^+^T cell migration assay

For in vitro assay of migrating CD8^+^ T cells, we utilized a transwell system with chambers featuring 5 μm-wide pores. After thorough washing and reconstitution in a serum-free medium, activated CD8^+^ T cells were introduced into the upper compartment of the culture chambers while adding a serum-containing medium to the lower compartment. Once the 24-h incubation was complete, cells were harvested from the lower section of the culture chambers before being quantified (Supplementary Fig. S2C).

### RNA extraction and real-time quantitative PCR

Total RNA extraction was performed utilizing TRIzol reagent (Ambion, 15596024), followed by the synthesis of cDNA through reverse transcription employing the PrimeScript RT kit (TaKaRa, Japan, RR037A). Quantitative real-time PCR (qRT-PCR) was performed using PowerUp^TM^ SYBR^TM^ Green Master Mix (Thermo Scientific^TM^, A25741), adhering to the guidelines provided by the manufacturer. Supplementary Table S2 lists details of the primer sequences.

### RNA-seq

To uncover the molecular basis of enhanced functionality and activity in PGRN^–/–^ CD8^+^ T cells in comparison to WT CD8^+^ T cells, we conducted a transcriptomic analysis using an in vitro culture model. Total RNA extraction was conducted by employing TRIzol reagent conforming to the manufacturer’s specific instructions. Subsequent to quality control, the VAHTS Universal V6 RNA-seq Library Prep kit was employed to create transcriptome libraries. Transcriptome sequencing and analysis were performed by the Shanghai Ouyi Biotechnology Co. The RNA-seq data in this study were deposited into NCBI databases under the accession number PRJNA1027261 (https://www.ncbi.nlm.nih.gov/sra/PRJNA1027261).

### Protein extraction and western blots

Cellular protein extraction was performed utilizing RIPA protein lysate, and protein concentrations were assessed using BCA quantification. Protein denaturation was achieved through 5-min boiling at 95 °C. For electrophoresis, 30 μg of the proteins was loaded and separated. After blocking with 10% skimmed milk, the membranes underwent incubation overnight at 4 °C with the primary antibody. Subsequently, on the following day, after being washed with TBST, these protein membranes underwent incubation for 1 h with the secondary antibody. Finally, chemiluminescence processing was performed on these membranes.

### Bioinformatics analysis

The RNA sequencing data (level 3), along with their clinical information, were acquired from 33 types of tumor patients through the Cancer Genome Atlas (TCGA) database (https://portal.gdc.com). The R packages ‘immuneconv’ were employed to perform a dependable immune correlation assessment, and all procedures were subjected to statistical analysis using R software v4.0.3.

### Statistical analysis

All experiments were repeated at least three times independently. The analysis of data was accomplished by utilizing the SPSS software (version 23.0). Unless specified otherwise, quantitative data are displayed as mean ± standard deviation (SD) for all measurements. Statistical differences were assessed using Student’s unpaired t-test for comparing two groups and one-way ANOVA followed by multiple comparison for multiple groups. The non-parametric Kruskal–Wallis test was employed for in vivo data. A threshold of *p* < 0.05 (*p < 0.05, **p < 0.01, ***p < 0.001, ****p < 0.0001) indicates statistical significance.

### Supplementary information

Supplementary Fig. Legends

supplemental figure S1

supplemental figure S2

supplemental figure S3

supplemental figure S4

supplemental figure S5

supplemental figure S6

supplemental figure S7

supplemental figure S8

Supplementary Table S1

Supplementary Table S2

Full and uncropped western blots

## Data Availability

Data supporting the findings of this study are available from the corresponding authors upon reasonable request.
