# Supplementary material for: Deciphering the potential role of PGRN in regulating CD8+ T cell antitumor immunity
Source: Cell Death Discov. 2024 May 14;10:233. doi: 10.1038/s41420-024-02001-7 (PMC11094002; doi:10.1038/s41420-024-02001-7)
Supplement: Supplementary file 1 — Supplementary Fig. Legends [file 41420_2024_2001_MOESM1_ESM.docx]

**Supplementary Figure Legends**

**Supplementary Figure S1.** A. Verification of the knockout efficiency of CD8^+^ T cells from PGRN-KO mice (n=6; ****p<0.0001). B. Subcutaneously transplanted tumors from WT and PGRN-KO mice. C. Flow cytometry analysis of tumor-infiltrating immune cell proportions, including CD45^+^ T, CD3^+^ T, and CD4^+^ T cells, M1 and M2 macrophage, NK cells, in addition to MDSCs (mean±SD; n=10/group; *p<0.05; ns nonsignificant). D. Representative immunofluorescence staining of CD8 (red) and DAPI (blue) in tumor tissues on days 7 and 17 post-subcutaneous tumor cell injection in mice; scale bar = 50 μm.

**Supplementary Figure S2.** A. Differential expression of PGRN in CD8^+^ T cells from the spleen and tumor:  Lewis cells underwent subcutaneous injection into the flanks of WT mice; subsequent to 21 days, CD8^+^ T cells from spleens as well as CD8^+^ TILs underwent sorting using FACS. RT-PCR was deployed to analyze mRNA expression levels of PGRN. Statistical analyses using the t-test (n=6; **p<0.01; ***p<0.001). B. Purity verification of the CD8^+^ T cells isolated from mouse spleens via magnetic bead sorting using flow cytometry. C. The construction process of the migration assay model of in vitro CD8^+^ T cells. D. In vitro CD8^+^ T cell cytotoxicity assay. E. Statistical analysis of DEGs in RNA-seq results: The horizontal axis represents the grouping of PGRN-KO and WT groups, while the vertical axis signifies the number of significant DEGs between the two groups. "Up" and "Down" indicate the count of significantly upregulated and downregulated genes, respectively.

**Supplementary Figure S3.** Gating strategy evaluation for the tumor-infiltrating immune cells: Fixable Viability Stain 700 distinguishes between dead and live cells. Various immune cell populations within the live cell gates were identified using a combination of specific antibodies. The presence of CD8^+^CD3^+^CD45^+^ markers was used to identify cells as CD8^+^ T cells. Based on the expression of surface molecules, including cytotoxicity markers (IFN-γ and GZMB), exhaustion markers (PD-1), proliferation markers (Ki67), and apoptosis markers (Annexin V/PI), several functional states of CD8^+^ T cells were identified. The CD4^+^ T cells were identified as CD4^+^CD3^+^CD45^+^ cells. M2-macrophages were defined as F4/80^+^CD11b^+^CD206^+^ cells. M1-macrophages were marked as F4/80^+^CD11b^+^CD86^+^ cells. NK cells were recognized as NK1.1^+^CD3^–^ cells, and MDSCs were identified as LY6G^+^/LY6C^+^CD11b^+^ cells. These gating strategies were developed using splenocytes obtained from the mice in the control group.

**Supplementary Figure S4.** PGRN knockout increases the expression of T cell chemokine CCL3. A. The volcano plot showcases genes with altered expression, known as "differentially expressed genes (DEGs)", as a result of PGRN knockout, including cytotoxic factors and chemokines. B. The heatmap illustrates changes in the expression of chemokines and cytotoxic factors influenced by PGRN knockout. C. Bubble chart of signaling pathways affected by PGRN knockout. The horizontal axis in the figure represents the enrichment score; larger bubbles correspond to entries with more genes encoding differential proteins. As the enrichment p-value decreases and the significance level rises, the color of the bubble changes from blue to white to yellow to red. A p-value <0.05 indicates significant enrichment. D. Gene Set Enrichment Analysis (GSEA) of the cytokine-cytokine receptor interaction pathway. E. Comparative analysis plot displaying chemokine gene expression levels measured in FPKM from RNA sequencing data (*p<0.05, **p<0.01, ****p<0.0001). F. The mRNA expression levels of specified genes in WT and PGRN-KO CD8^+^ T cells were quantified using RT-PCR. Statistical analyses were conducted through the utilization of a t-test (n=3; *p<0.05; ***p<0.001; ****p<0.0001; ns nonsignificant). G. The CCL3 protein exhibited elevated levels in the PGRN^–/–^ CD8^+^ T cells when Western blotting was employed (n=3).

**Supplementary Figure S5.** CCL3-mediated enhanced in vitro antitumor immunity caused by PGRN knockout. A. The treatment led to changes in the proportion of CD8^+^ T cells undergoing apoptosis (n=3/group; **p<0.01, ****p<0.0001). By employing flow cytometry analysis, identification of expression levels of (B) cytotoxicity markers IFN-γ and (C) GZMB, in addition to (D) exhaustion marker PD-1 in the CD8^+^ T cells in all groups was conducted (n=3/group; ****p<0.0001 for IFN-γ and GZMB; ***p<0.001, ****p<0.0001 for PD-1). E. The treatment led to changes in the proportion of CD8^+^ T cells at different stages of the cell cycle (n=3/group; **p<0.01; ns nonsignificant). F. Treatment-induced alterations in the population of Ki67^+^ CD8^+^ T cells (n=3/group; ****p<0.0001). G. Changes in CD8^+^ T cell migration in vitro (n=3/group; ****p<0.0001). H. P815 cells underwent co-culture with pre-activated CD8^+^ T cells at a 1:10 ratio without or with an anti-CCL3 neutralizing antibody for 1 day. Apoptotic P815 cell percentage was evaluated by employing the flow cytometry technique in an apoptosis assay (****p<0.0001).

**Supplementary Figure S6.** CD8^+^ T cells from patients with lung cancer express higher levels of PGRN compared to healthy individuals. A. PGRN expression in CD8^+^ T cells from both healthy individuals and lung cancer patients (n=5/group; **p<0.01). B. The mRNA levels of PGRN and CCL3 were analyzed using RT-PCR in CD8^+^ T cells sorted by FACS from PBMCs of both healthy individuals and lung cancer patients. Statistical analyses were conducted through the utilization of a t-test (n=5/group; **p<0.01).

**Supplementary Figure S7.** A. Correlation heatmap of PGRN and other classical immune checkpoint expression levels in different tumor tissues. B. Correlation heatmaps of PGRN and multiple immune cell infiltration scores in different tumor tissues.

**Supplementary figure S8.** A. Western blot analysis revealed elevated levels of the PGRN protein in CD8^+^TILs isolated from the TME of WT tumor-bearing mice (n=3). B. Western blot analysis showed a significant decrease in PGRN protein levels in PGRN-KO tumors compared to those in WT mice (n=3).
