# Supplementary figures and images for: Deciphering the potential role of PGRN in regulating CD8+ T cell antitumor immunity
Source: Cell Death Discov. 2024 May 14;10:233. doi: 10.1038/s41420-024-02001-7 (PMC11094002; doi:10.1038/s41420-024-02001-7)

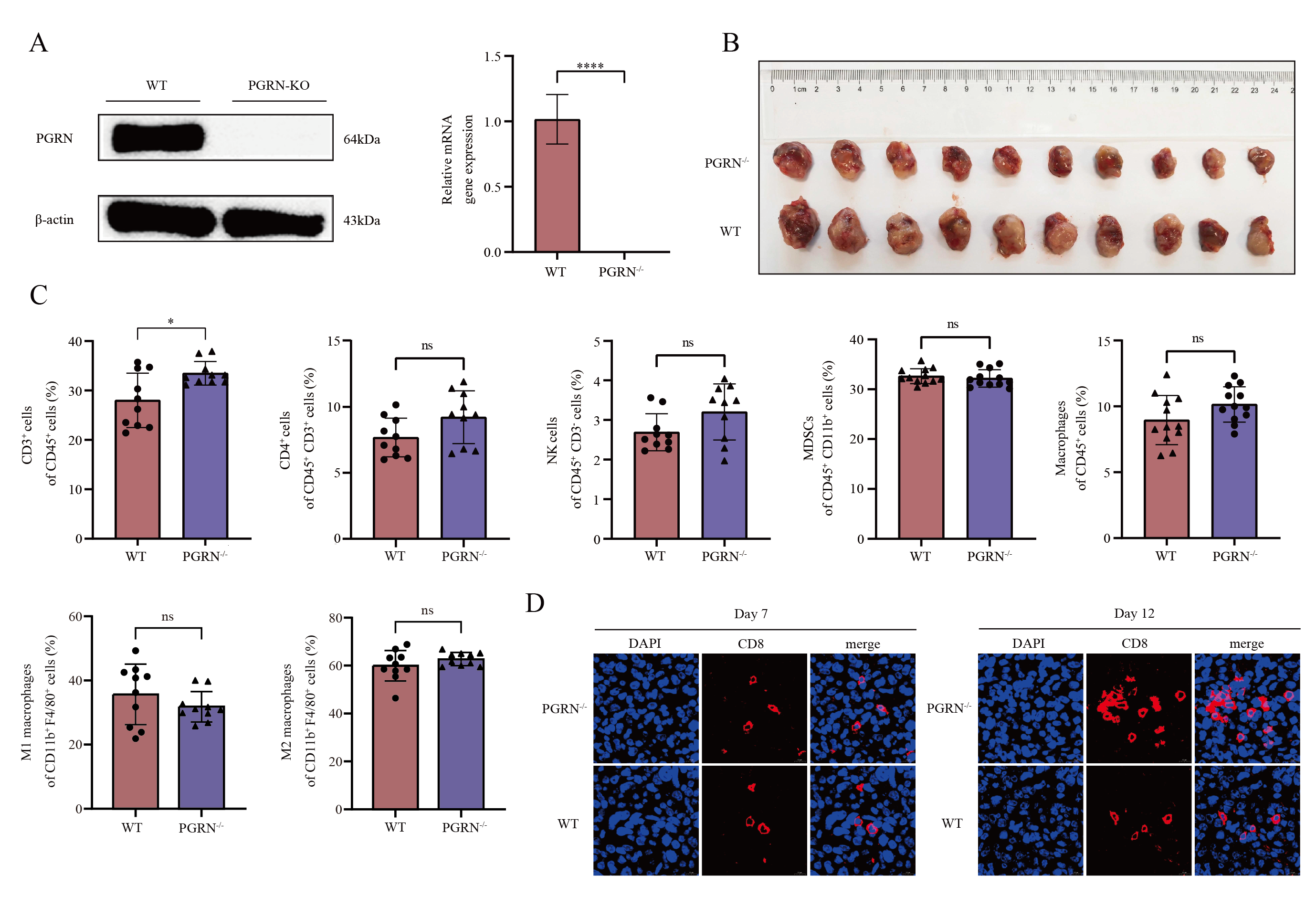

Supplement: Supplementary file 2 — supplemental figure S1 [file 41420_2024_2001_MOESM2_ESM.png]

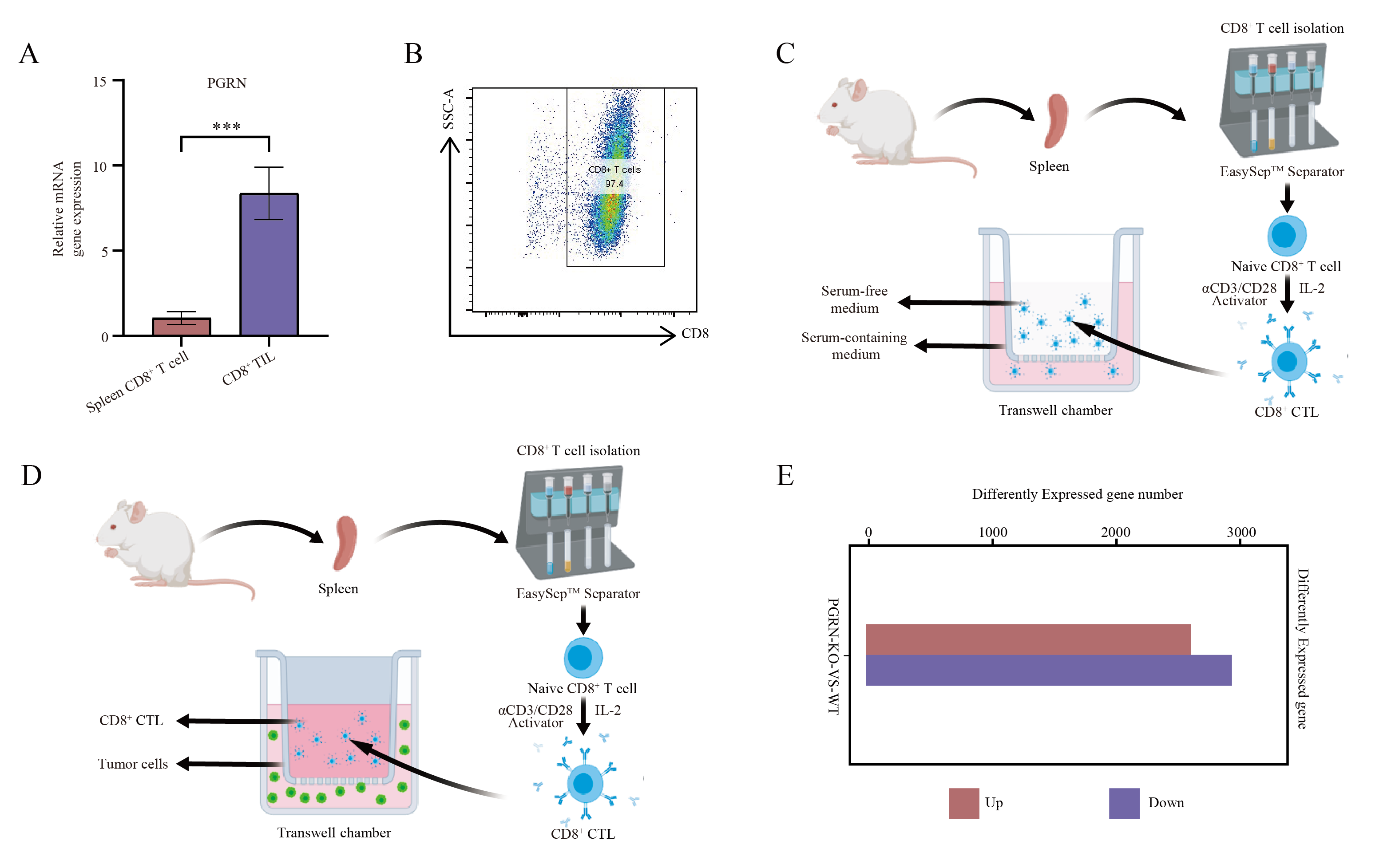

Supplement: Supplementary file 3 — supplemental figure S2 [file 41420_2024_2001_MOESM3_ESM.png]

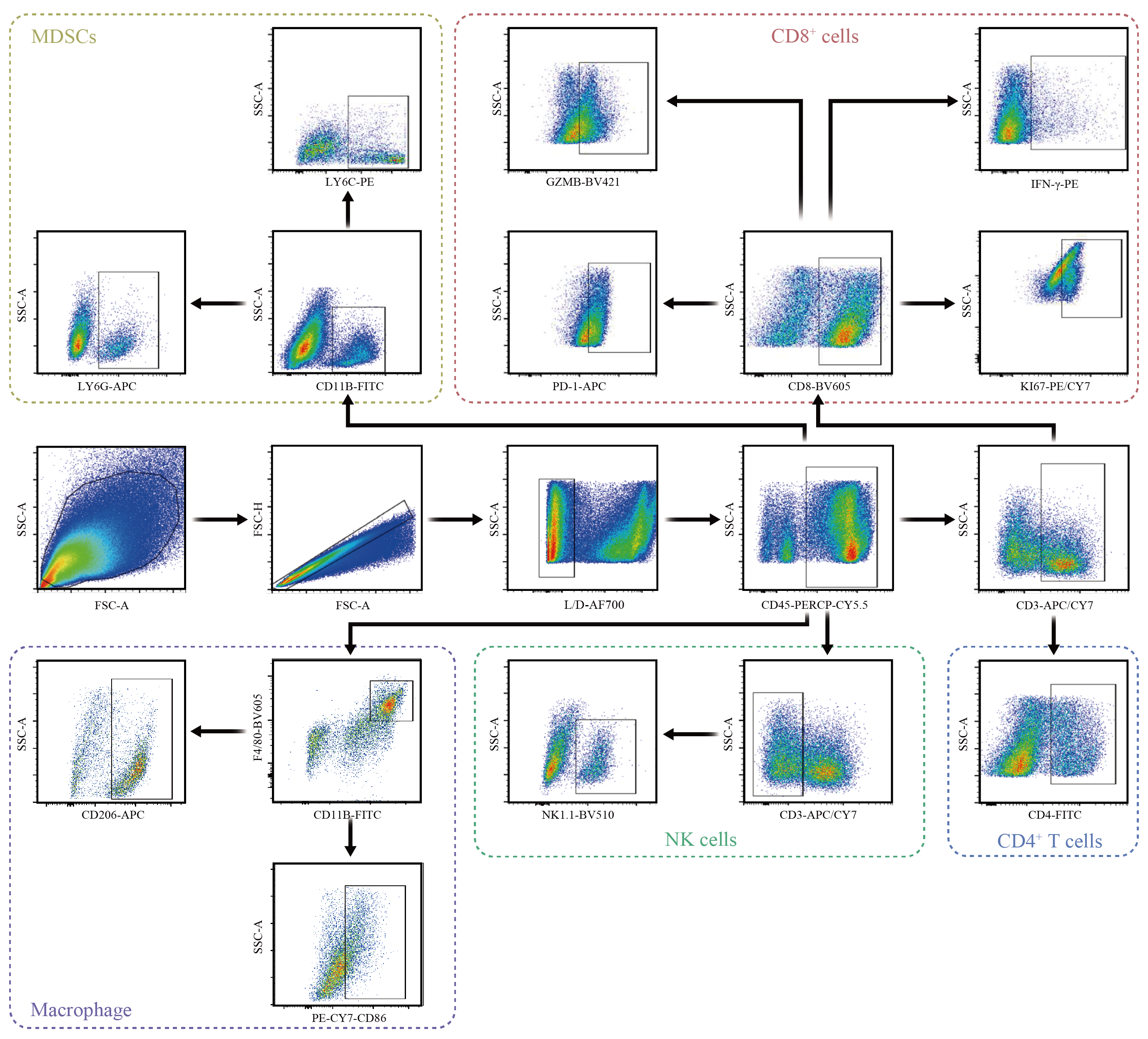

Supplement: Supplementary file 4 — supplemental figure S3 [file 41420_2024_2001_MOESM4_ESM.png]

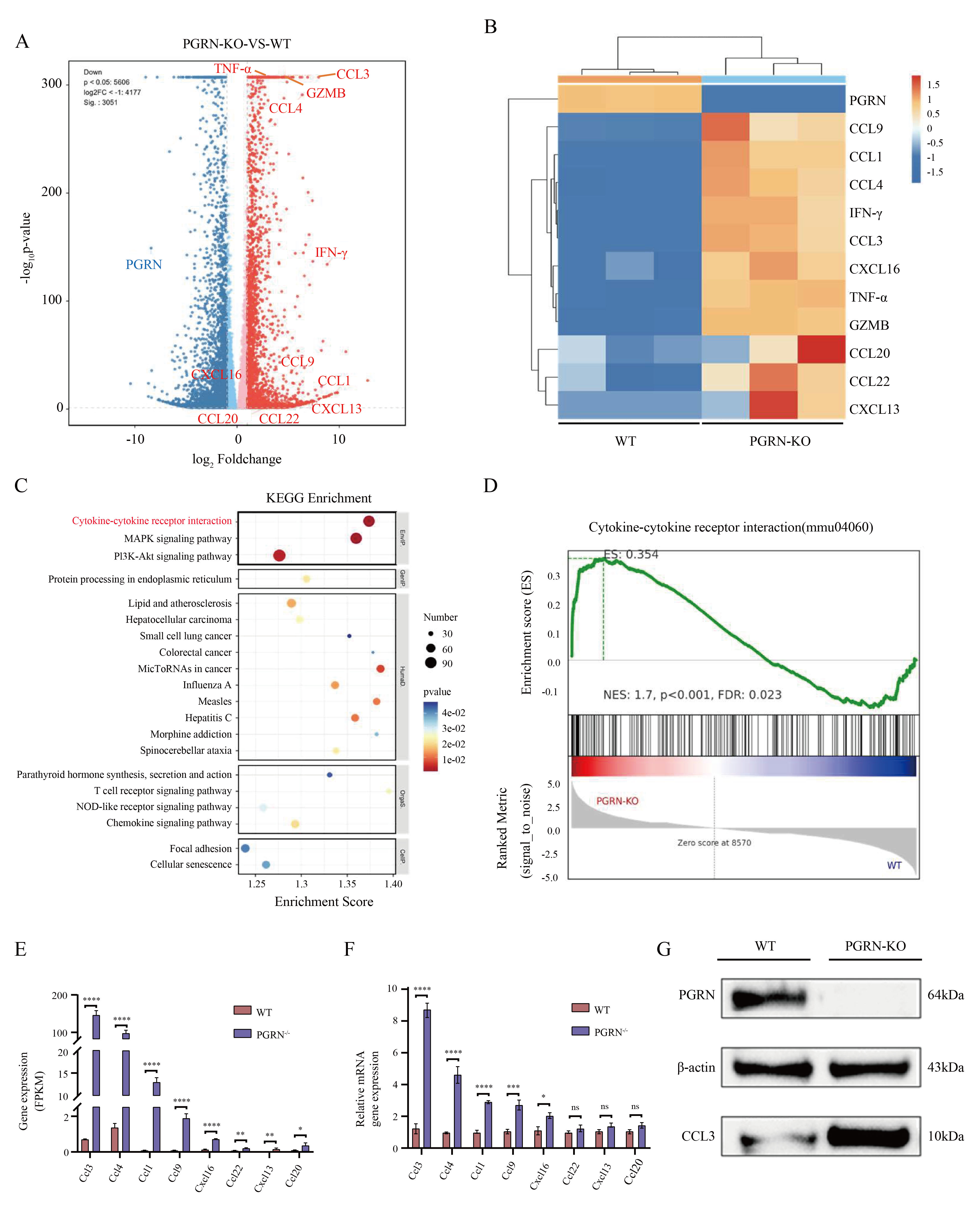

Supplement: Supplementary file 5 — supplemental figure S4 [file 41420_2024_2001_MOESM5_ESM.png]

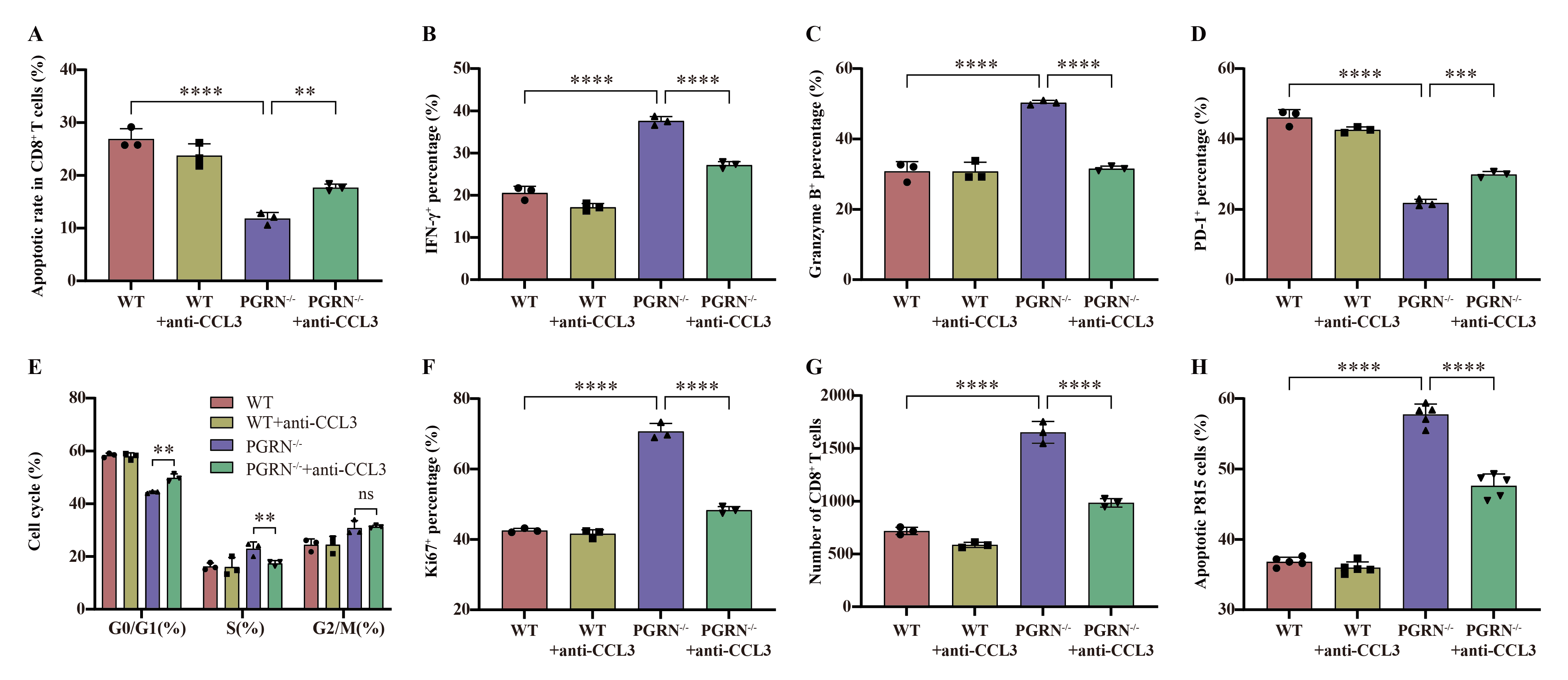

Supplement: Supplementary file 6 — supplemental figure S5 [file 41420_2024_2001_MOESM6_ESM.png]

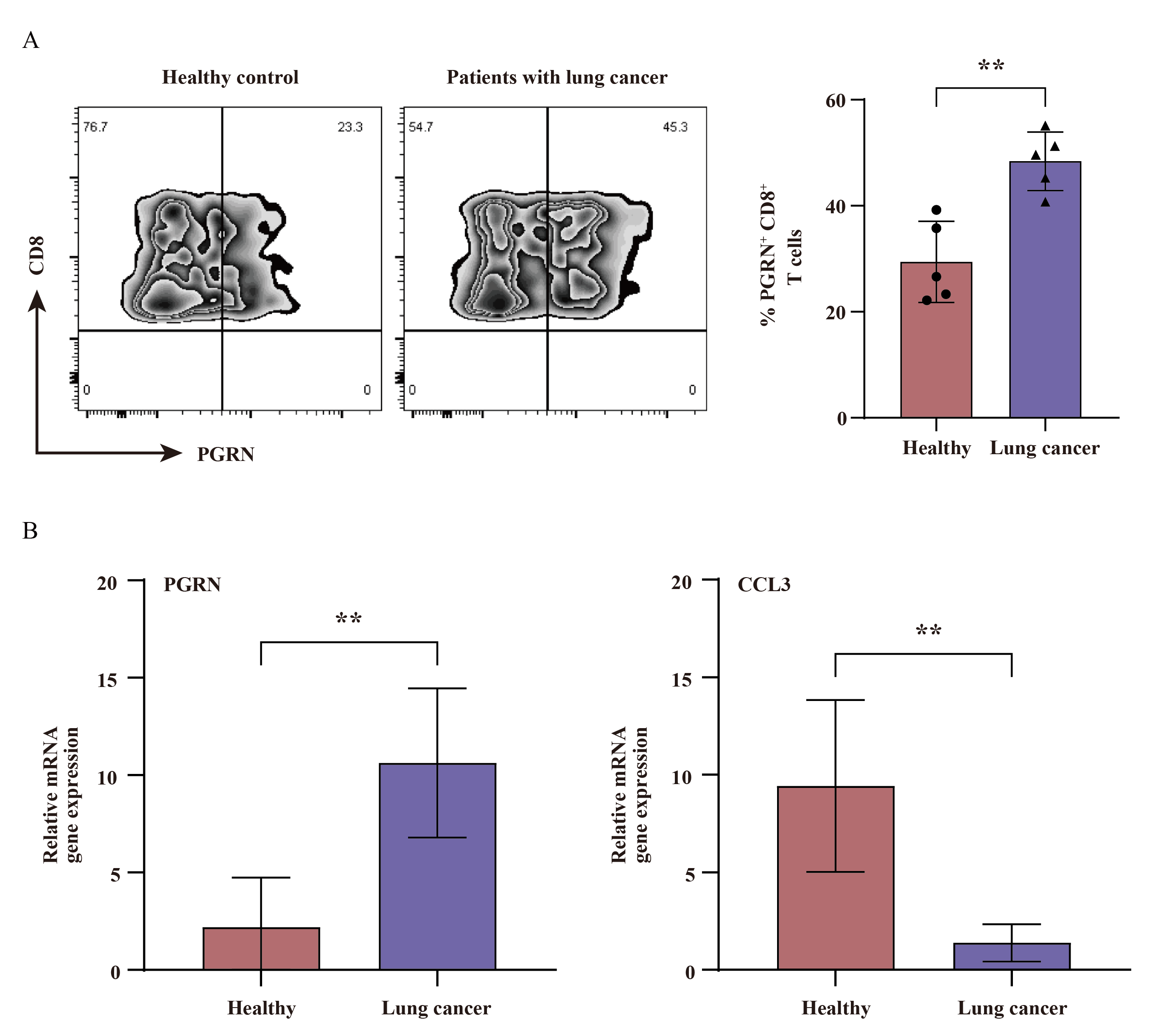

Supplement: Supplementary file 7 — supplemental figure S6 [file 41420_2024_2001_MOESM7_ESM.png]

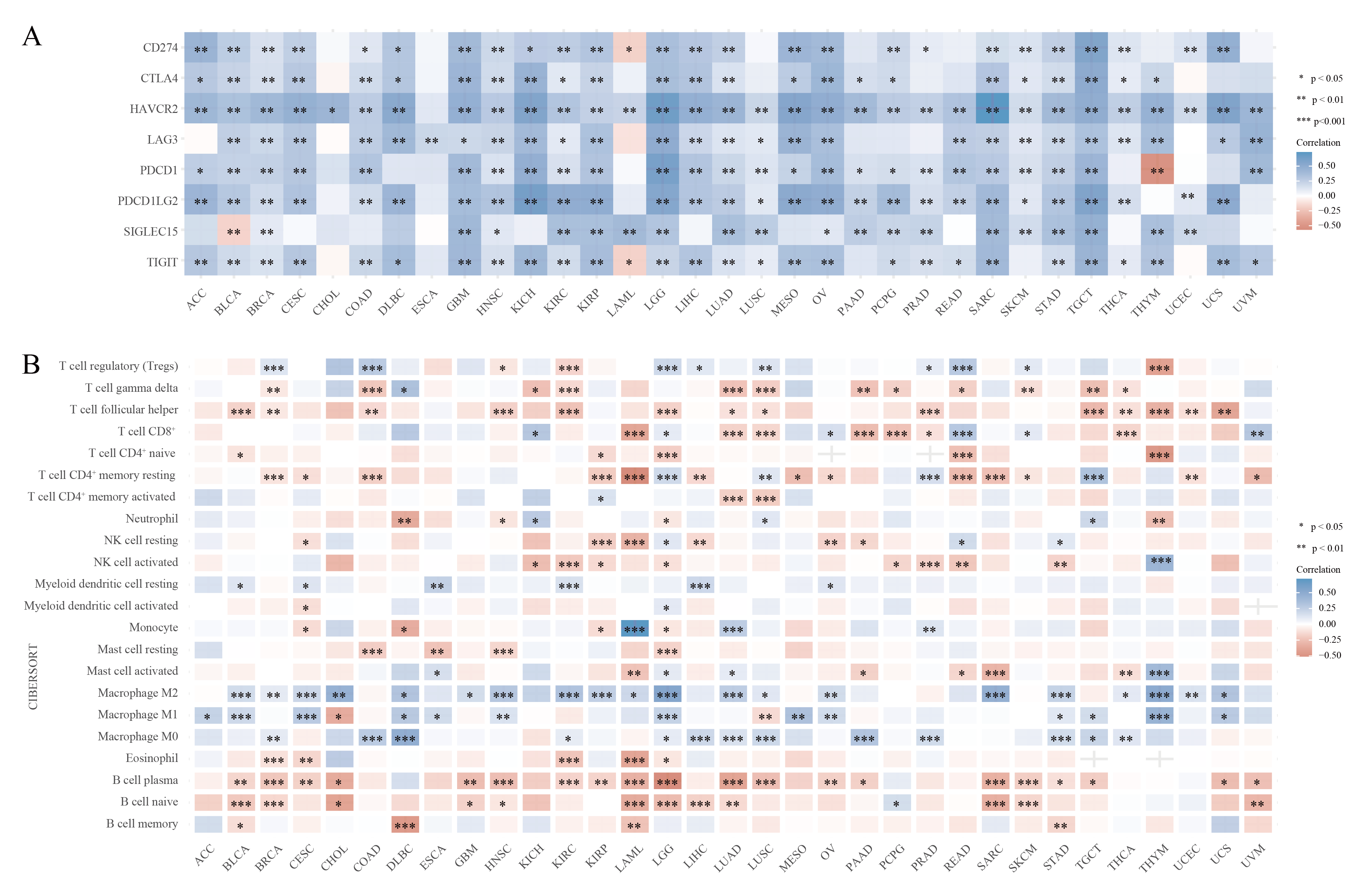

Supplement: Supplementary file 8 — supplemental figure S7 [file 41420_2024_2001_MOESM8_ESM.png]

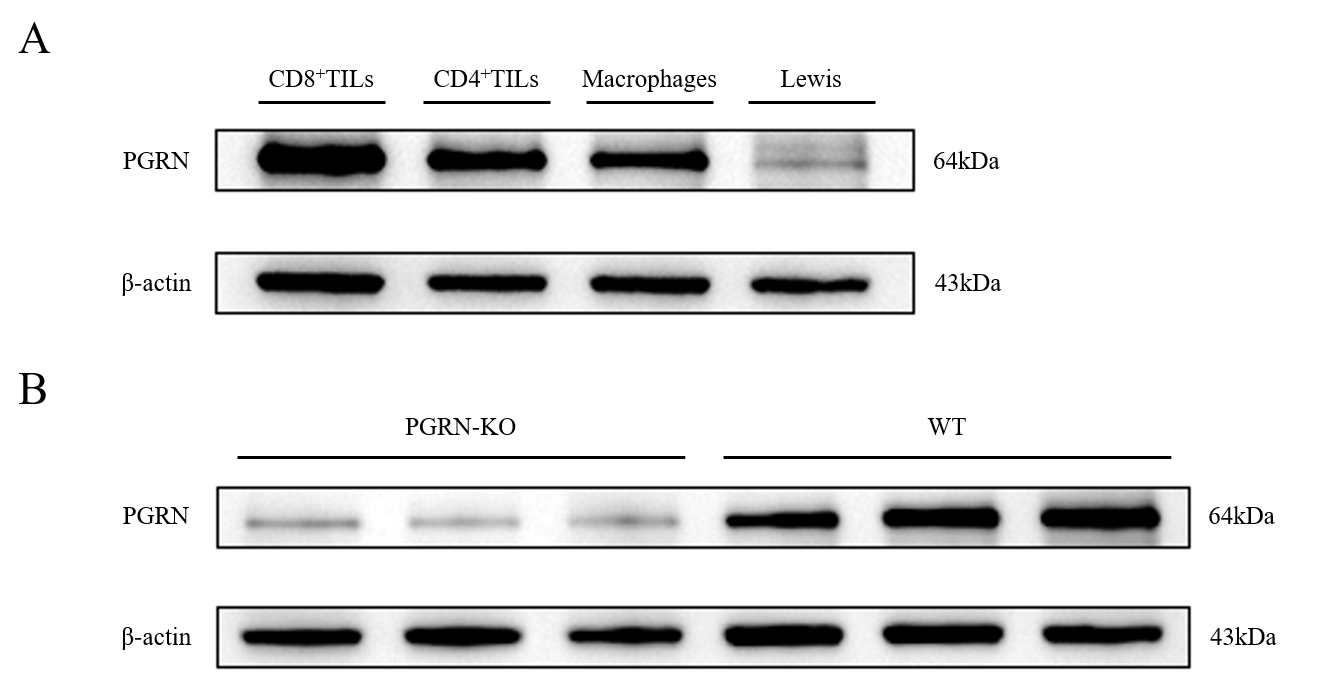

Supplement: Supplementary file 9 — supplemental figure S8 [file 41420_2024_2001_MOESM9_ESM.png]

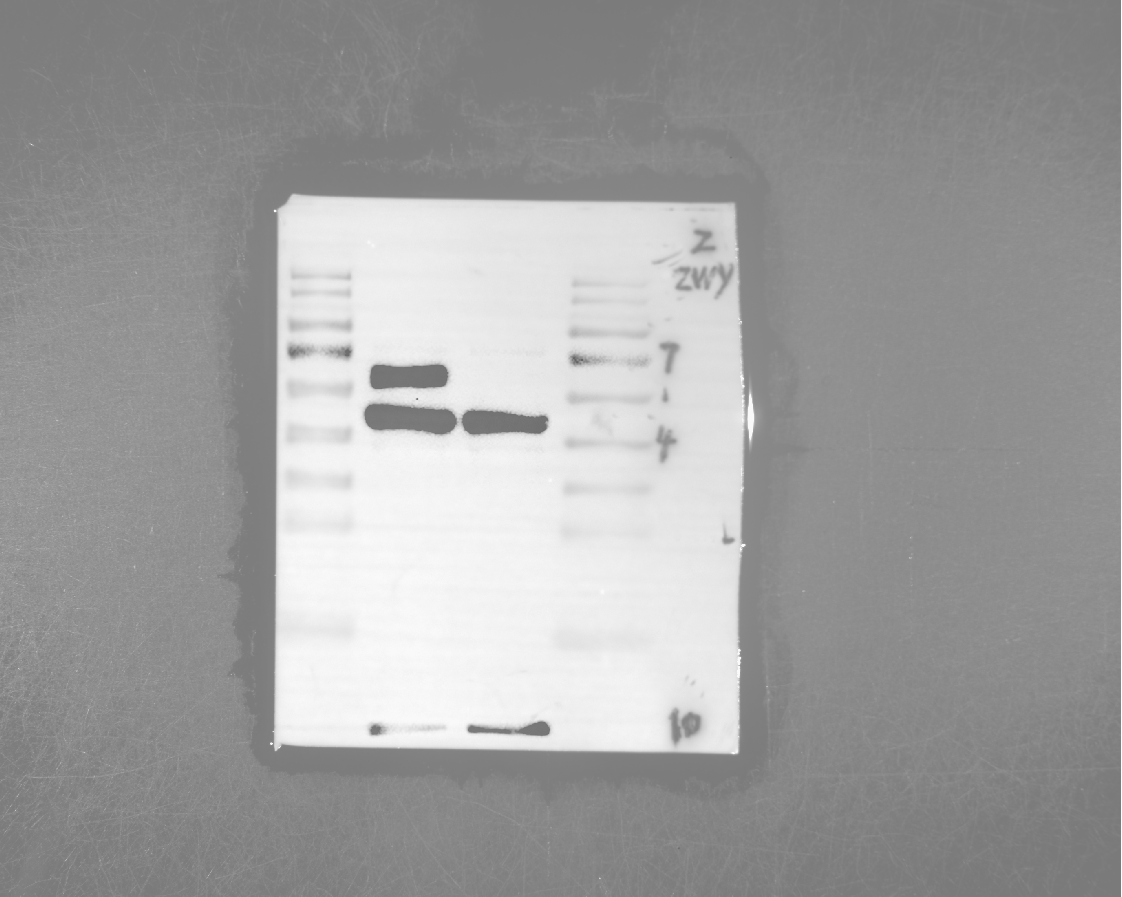


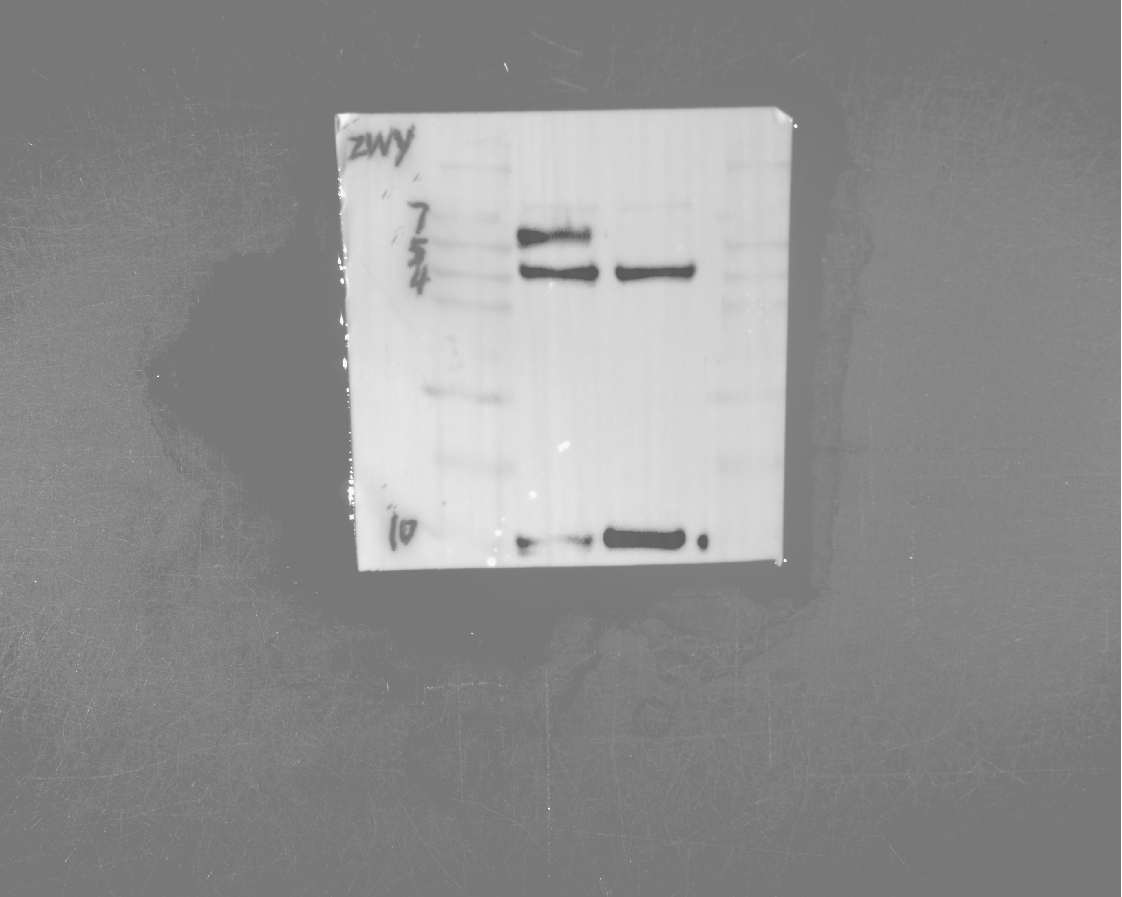


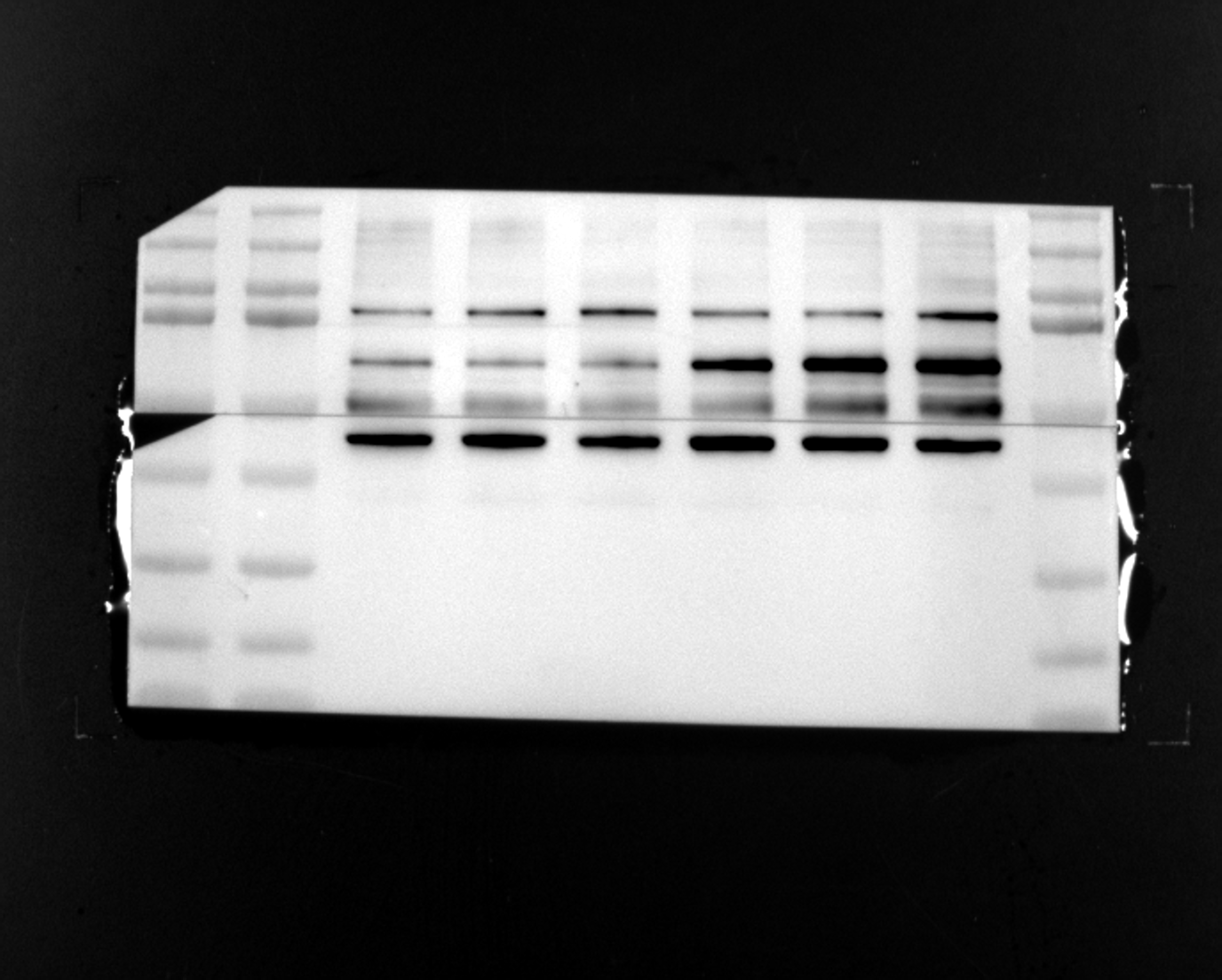


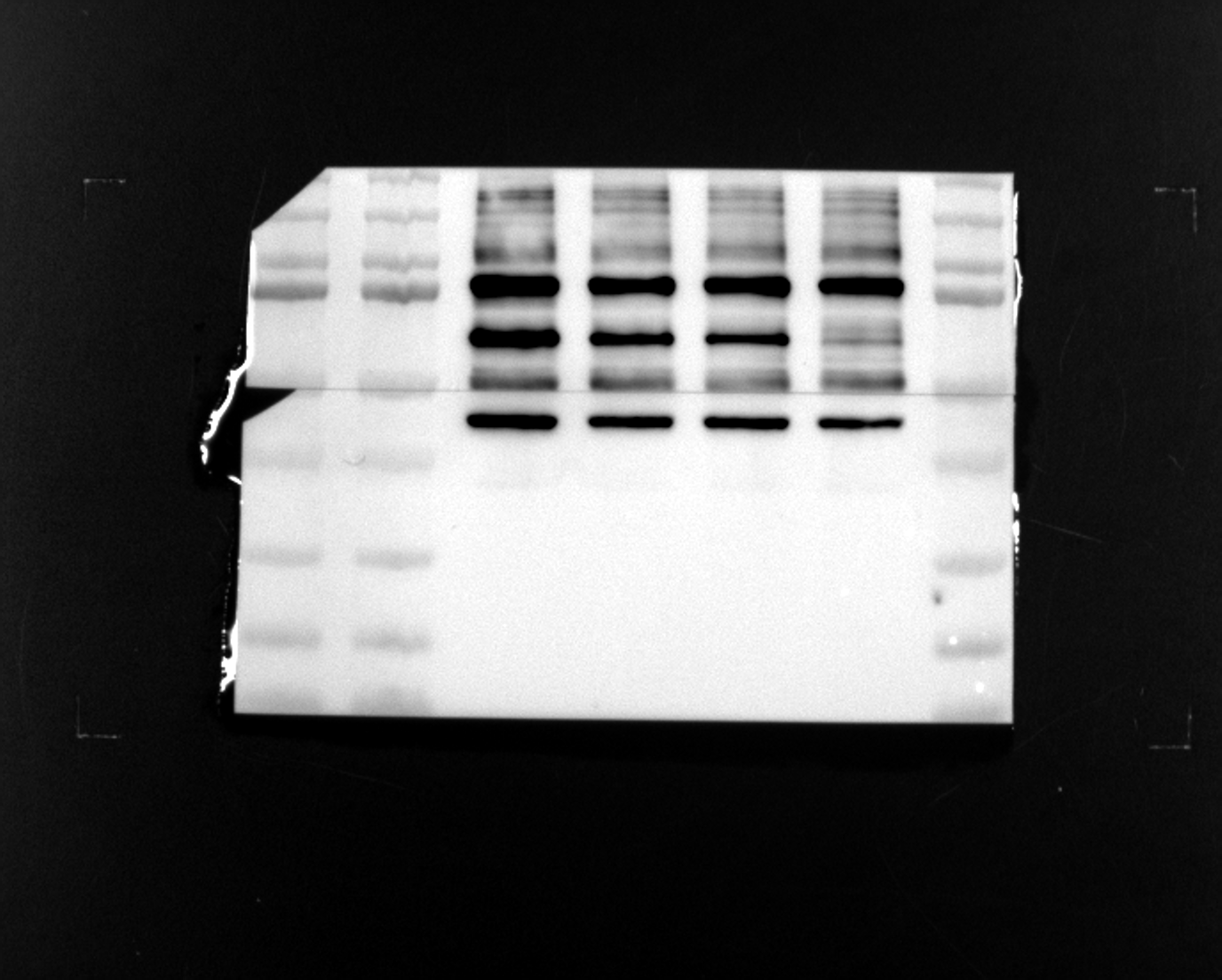

Supplement: Supplementary file 12 — Full and uncropped western blots [file 41420_2024_2001_MOESM12_ESM.doc]
