# Supplementary material for: Deciphering the potential role of PGRN in regulating CD8+ T cell antitumor immunity
Source: Cell Death Discov. 2024 May 14;10:233. doi: 10.1038/s41420-024-02001-7 (PMC11094002; doi:10.1038/s41420-024-02001-7)
Supplement: Supplementary file 10 — Supplementary Table S1 [file 41420_2024_2001_MOESM10_ESM.docx]

**Supplementary Table S1.** List of key materials and reagents.

| **REAGENT** | **SOURCE** | **IDENTIFIER** |
| --- | --- | --- |
| RPMI-1640 | Gibco | 2183707 |
| 2-mercaptoethanol | Gibco | 2188984 |
| HEPES | Gibco | 2152913 |
| Penicillin-Streptomycin | Gibco | 2240831 |
| Heat inactivated fetal bovine serum | Gibco | 2280547cp |
| Fixable Viability Stain 700 | BioLegend | 564997 |
| PERCP-CY5.5 anti-mouse CD45 | BD Pharmingen™ | 550994, |
| APC-CY7 anti-mouse CD3 | Biolegend | 557596 |
| FITC anti-mouse CD4 | Biolegend | 553046 |
| BV650 anti-mouse CD8a | BioLegend | 100751 |
| PE anti-mouse LY-6C | Thermo Fisher | 12-5932-82 |
| APC anti-mouse LY-6G | Thermo Fisher | 17-9668-82 |
| BV510 anti-mouse NK1.1 | Biolegend | 563096 |
| FITC anti-mouse CD11b | BioLegend | 557396 |
| EF450 anti-mouse GZMB | Thermo Fisher | 48-8898-82 |
| APC anti-mouse CD278(PD-1) | BioLegend | 562671 |
| PE anti-mouse IFN-γ | BioLegend | 554412 |
| BV605 anti-mouse F4/80 | BioLegend | 743281 |
| PE anti-mouse Ki-67 | Biolegend | 567719 |
| rabbit anti-PGRN (1:1000) | Abcam | ab191211 |
| mouse anti-GAPDH (1:3000) | R&D SYSTEMS | 2275-PC-100 |
| EasySep Mouse CD8^+^T cell Isolstion Kit | Stemcell | 19853 |
| InVivoMAb anti-mouse CD3ε | BioXCell | BE0001-1 |
| InVivoMAb anti-mouse CD28 | BioXCell | BE0015-1 |
| Recombinant Mouse IL-2 | R&D SYSTEMS | 575406 |
| Anti-PD-L1 monoclonal antibody | BioXCell | 10F.9G2 |
| IgG2b isotype control | BioXCell | BE0090 |
| Anti-CD8 neutralizing antibody | BioXCell | BE0061 |
| Anti-CCL3 neutralizing antibody | R&D Systems | AF-450-NA |
| CD8 (1:300) | Servicebio | GB21301 |
| Tumor Dissociation Kit | Miltenyi | 130-096-730 |
| Cell Cycle Assay Kit | Abcam | ab112117 |
| PowerUpTM SYBRTM Green Master Mix | Thermo Scientific™ | A25741 |
| RevertAid Fist Strand cDNA Synthesis KIT | Thermo Scientific™ | 00799576 |
| Goat anti-Rabitt Secondary antibody | Servicebio | GB21303 |
| Goat anti-Rabitt Secondary antibody | Servicebio | GB25303 |
| DAPI | Servicebio | G1012 |
