# Supplementary material for: Deciphering the potential role of PGRN in regulating CD8+ T cell antitumor immunity
Source: Cell Death Discov. 2024 May 14;10:233. doi: 10.1038/s41420-024-02001-7 (PMC11094002; doi:10.1038/s41420-024-02001-7)
Supplement: Supplementary file 11 — Supplementary Table S2 [file 41420_2024_2001_MOESM11_ESM.docx]

**Supplementary Table S2.** Sequences of primers used for qRT-PCR.

| **Gene name** | **Forward (5′-3′)** | **Reverse (5′-3′)** |
| --- | --- | --- |
| PGRN | TGCCATGATAACCAGACCT | GCAACAGTGACGTCCATC |
| GranzymeB | CCACTCTCGACCCTACATGG | GGCCCCCAAAGTGACATTTATT |
| Bcl-xL | TGGTGGTCGACTTTCTCTCC | CTCCATCCCGAAAGAGTTCA |
| Gapdh | TGGATTTGGACGCATTGGTC | TTTGCACTGGTACGTGTTGAT |
| IFN-γ | GATGCATTCATGAGTATTGCCAAGT | GTGGACCACTCGGATGAGCTC |
| TNF-α | AATGGCCTCCCTCTCATCAGT | GCTACAGGCTTGTCACTCGAATT |
| CCL20 | GAAGCAGCAAGCAACTACGAC | GTTCACAGCCCTTTTCACCC |
| OX-40 | AACCTCGGCAGGACAGCGGC | CACTGGCTGGGTGGCGGGTC |
| 4-1BB | CCCCCACATATTCAAGCAAC | TAGCCTCCTCCTCCTCCTTC |
| CCL3 | TTCTCTGTACCATGACACTCTGC | CGTGGAATCTTCCGGCTGTAG |
| CCL4 | TTCCTGCTGTTTCTCTTACACCT | CTGTCTGCCTCTTTTGGTCAG |
| CCL1 | GGCTGCCGTGTGGATACAG | AGGTGATTTTGAACCCACGTTT |
| CCL9 | CCCTCTCCTTCCTCATTCTTACA | AGTCTTGAAAGCCCATGTGAAA |
| CCL22 | AGGTCCCTATGGTGCCAATGT | CGGCAGGATTTTGAGGTCCA |
| CXCL16 | CCTTGTCTCTTGCGTTCTTCC | TCCAAAGTACCCTGCGGTATC |
| CXCL13 | GGCCACGGTATTCTGGAAGC | GGGCGTAACTTGAATCCGATCTA |
